# Sociodemographic changes in the population frequency of colonoscopy following the implementation of organised bowel cancer screening: An analysis of data from Swedish registers, 2006–2015

**DOI:** 10.1177/0969141320957708

**Published:** 2020-09-22

**Authors:** Torbjörn Thulin, Ulf Strömberg, Anders Holmén, Rolf Hultcrantz, Anna Forsberg

**Affiliations:** 1 Department of Medicine, Karolinska Institutet, Solna, Sweden; 2 Department of Research and Development, Region Halland, Halmstad, Sweden; 3 School of Public Health and Community Medicine, Institute of Medicine, Sahlgrenska Academy at University of Gothenburg, Gothenburg, Sweden

**Keywords:** Colorectal cancer, CRC screening, colonoscopy, epidemiology

## Abstract

**Objective:**

To assess sociodemographic changes in the population frequency of colonoscopy (PFC; number of colonoscopies per 1000 inhabitants per year) among people aged 50–74 in relation to the implementation of a regional colorectal cancer screening programme for people aged 60–69 in the Stockholm-Gotland region (RSG) in 2008.

**Method:**

The PFC was estimated by year (2006–2015), pre- and post-implementation of colorectal cancer screening programme (2006–2007 vs. 2014–2015), age, sex, residential region, immigrant status and educational level. The data were obtained from Swedish patient and population registers.

**Results:**

The PFC largely increased during 2006–2015 in all six Swedish regions. The estimated increase in the pre- vs. post period PFC (ΔPFC) within the RSG was (i) greater for men than for women (5.8 vs. 4.5) and (ii) smaller for people aged 70–74 than for those aged 60–69 (5.5 vs. 9.0), while the corresponding ΔPFCs within each of the other regions were (i) not greater, or even smaller, for men and (ii) not smaller, or even larger, for elderly people aged 70–74.

**Conclusion:**

A regional implementation of an organised colorectal cancer screening programme did not lead to a higher PFC increase in the screening relevant age group 50–74 years. Nevertheless, changes in the PFC were more pronounced for men and less pronounced for people aged 70–74 than those invited to participate in the screening programme (60–69 years), as compared with the rest of Sweden (without organised colorectal cancer screening).

## Introduction

The European guidelines for quality assurance in colorectal cancer (CRC) screening and diagnosis recommend screening between the ages of 50 and 74 years.^1^ Notwithstanding, people from younger age groups can also be considered for screening. Recent guidelines from the American Cancer Society recommend lowering the starting age for routine CRC screening to 45 years, as the incidence of CRC has risen in younger adults.^2^ Data on the effectiveness of screening in elderly people have provided support for the upper age limit to be set at 74 years.^3^ The commonly applied tests for CRC screening are stool-based tests to detect blood and endoscopic methods, either sigmoidoscopy or colonoscopy. In recent years, the faecal immunochemical test (FIT) is the predominantly used stool-based test.^4^ FIT screening is generally associated with higher participation and detection rates of adenomas and CRC compared with the more previously used guaiac faecal occult blood test (FOBT).^5^,^6^ FOBT screening and the impact of the test are limited by the poor-to-moderate sensitivity for advanced adenomas and cancers.^7^ Endoscopy can be used as both a primary screening tool and a follow-up examination for individuals who have tested positive via another method. Colonoscopy is expected to be more effective than sigmoidoscopy because it reaches the whole large bowel, not only the distal part.^8^ In contrast to opportunistic screening, organised screening involves actively inviting all individuals within predefined age intervals who live in a specific area to take part in a protocol-based screening.

The correlation between socioeconomic status (SES) and health status is fairly undisputed. SES impacts survival for several groups of cancers, including colorectal malignancies.^9^,^10^ Only about half of the organised screening programmes worldwide collect data on participation by SES and ethnicity, 90% of which report lower participation among lower socioeconomic groups.^11^ Mortality rates from CRC have declined considerably in recent decades as screening programmes have been implemented, but reductions in mortality and increased survival rates have reportedly been greater in the population groups with a high SES.^10–13^ Men have higher CRC incidence rates and mortality rates than women.^14^ Despite that, several studies have shown that the uptake in FIT screening programmes is lower among men. A recent meta-analysis reported a significantly lower uptake by men vs. women (odds ratio: 0.84; 95% confidence interval: 0.75–0.95; *P* < 0.01).^15^ An organised CRC screening programme may therefore potentially create or exacerbate sociodemographic inequities in health care and health outcomes.

We aimed to study the changes in the *population frequency of colonoscopy* (PFC; number of colonoscopies per 1000 inhabitants per year) among individuals aged 50–74 in relation to the regional implementation of a CRC screening programme in Sweden, inviting 60–69-year-old people for a stool-based blood test. One of the six health regions in Sweden, the region of Stockholm-Gotland (RSG), started to implement such a screening programme in 2008. In the first years of the RSG screening programme, guaiac-based FOBT was used, and later FIT was introduced. As at 2015, the other five regions had not implemented organised screening. In this study, we assessed how the implementation of this organised CRC screening programme affected PFC in a screening-relevant population (50–74 years) in the RSG in comparison with the relevant control areas. The proportion of colonoscopies that stemmed from the organised CRC screening programme out of the total number of registered colonoscopies in the RSG among 50–74-year-old individuals from the start in 2008 up to 2015 was approximately 15%. We hypothesised that this proportion would have an impact on the sociodemographic distribution of all colonoscopies among people in a screening-relevant age group. We aimed to provide new information, based on real-life data, of how an organised CRC screening programme in a predominately public-financed health-care system reallocates colonoscopy resources in a screening-relevant population. To our knowledge, from a population perspective, the impact of organised CRC screening on colonoscopy frequency has not been previously studied.

## Materials and methods

### Study population

**Figure 1.**
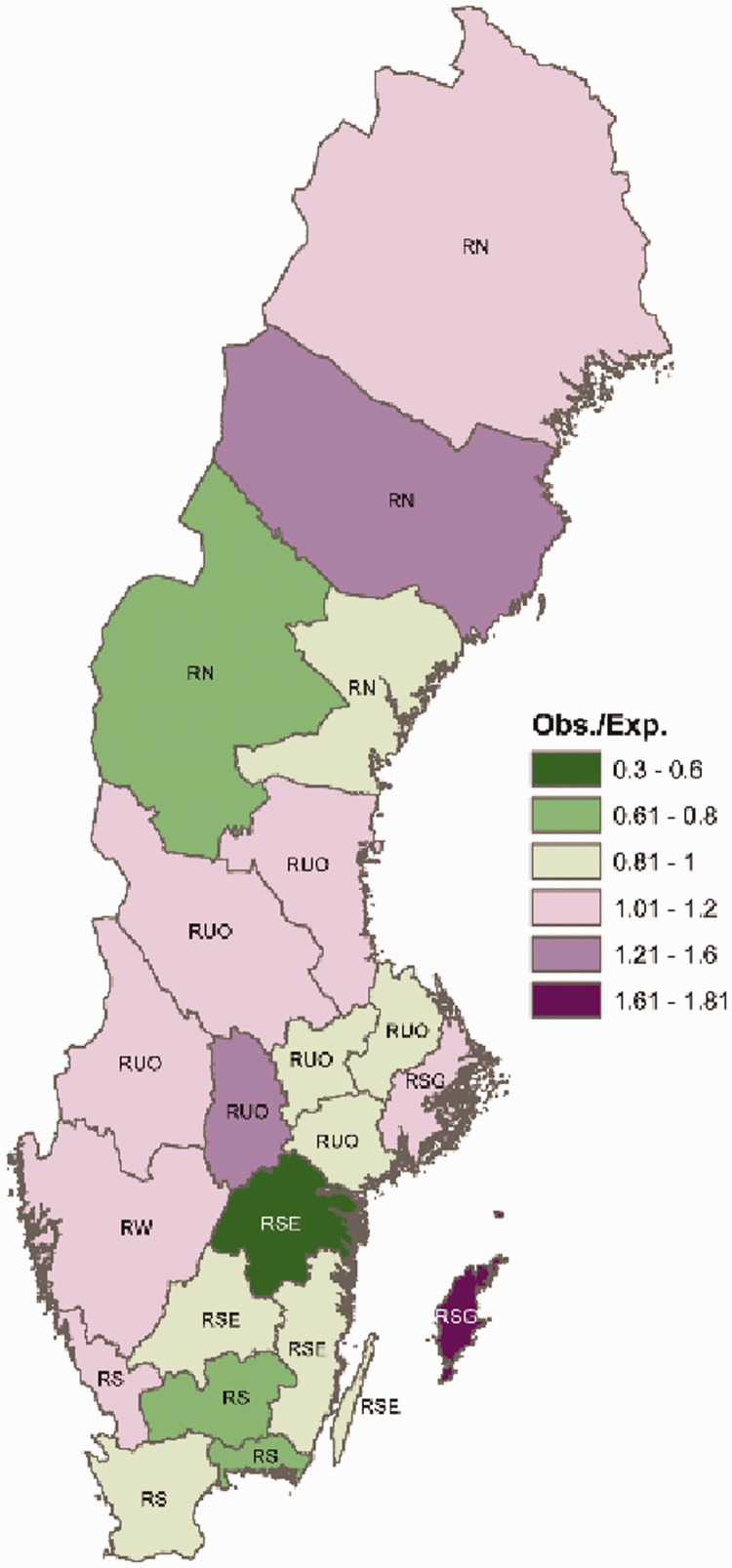
A map of Sweden displaying the observed-to-expected number of colonoscopies in each of the 21 counties (2006–2015). The labels for each county indicate which region a county belongs to (RSG, Region Stockholm-Gotland; RW, Region West; RS, Region South; RSE, Region South-East; RUO, Region Uppsala-Örebro; RN, Region North).

This study comprised the Swedish population aged 50–74 for the period 2006–2015. We studied sociodemographic changes with regard to residential region, calendar year, sex, age and immigrant status in combination with educational level, using population-based register data. We compared the PFC patterns for the period 2006–2015 between the RSG and each of the other five health-care regions. These five regions are Region West (RW), Region South (RS), Region South-East (RSE), Region Uppsala-Örebro (RUO) and Region North (RN) (Figure 1). The population data were stratified by region, sex, age group (50–54, 55–59, 60–64, 65–69 and 70–74 years) and country of birth (Swedish-born vs. foreign-born) in combination with educational level (data obtained from Statistics Sweden). Swedish-born individuals were classified based on the number of school years completed at the end of the year of diagnosis (“low” ≤9 years [primary school], “intermediate” = 10–12 years [high school/pre-university level] and “high” ≥13 years [university level]). We decided to disregard the registered information on educational level for foreign-born individuals because of considerable misclassification.^16^ Accordingly, four immigrant status/educational level groups were considered: Swedish-born with a (i) low, (ii) intermediate or (iii) high educational level, and (iv) foreign-born.

In 2006, there were 2,584,000 inhabitants between 50 and 74 years of age living in Sweden, with the following regional distribution: the RSG, 509,000; RW, 424,000; RS, 515,000; RSE, 286,000; RUO, 581,000; and RN, 270,000. In 2015, the corresponding population sizes were: Sweden, 2,887,000; the RSG, 609,000; RW, 476,000; RS, 574,000; RSE, 311,000; RUO, 633,000; and RN, 282,000.

### Patient data

The data on performed colonoscopies were collected retrospectively from the Swedish Hospital Discharge Register and the Swedish Outpatient Register for residents of Sweden older than 18 years, as previously described in detail by Forsberg et al.^17^ Using the codes for colonoscopy (UJF32 or UJF35), all registered colonoscopies performed in the period 2006–2015 for patients aged between 50 and 74 were included. Only one colonoscopy per person and year was included in the analysis. To obtain the proportion of colonoscopies generated from the RSG CRC screening programme, we gained access to the RSG regional quality CRC register. Within the present legislation, procedures performed by nurses are not recorded in the registers; therefore, data on nurse endoscopies were not available. Moreover, due to their various reimbursement methods, some private health-care providers do not report their colonoscopies to the national registries, which are therefore not included in the study.

Based on their unique Swedish identity numbers,^18^ the registered colonoscopy patients were linked to Statistics Sweden’s population registers. Data on country of birth and educational level were obtained from Statistics Sweden, and patients were classified in the same manner as the population, as described above.

### Statistical methods

Poisson regression^19^ was employed to estimate the PFC patterns and incorporated the following covariates: calendar year, age group, sex and immigrant status/education level (one model for each region). The PFC in a specified sociodemographic group was estimated by the corresponding model-based marginal mean. We paid particular attention to the changes in PFC between the pre- (2006–2007) and post-screening (2014–2015) periods in the RSG. This post-period was chosen with consideration for the successive development of the screening activity as from the initiation of the screening programme in 2008.^20^ The post–pre differences in PFC (ΔPFC) were estimated by suitable parameterisation of the Poisson regression models – with period (pre- and post-), age, sex and educational level/immigrant status as covariates (one model for each region). Adjustments for multiple comparisons were applied using the least significant difference method. The analyses were carried out using IBM Statistics for Windows version 25.0 (IBM Corp., Armonk, NY).

We produced a map of Sweden visualising the observed-to-expected number of colonoscopies in each county (Sweden comprises 21 counties) – where the expected number of colonoscopies was obtained from the year-, age- and sex-specific PFC for the total study population. More precisely, the expected number of colonoscopies was calculated as follows: For a given county, let P*_i,j,k_*denote the population size stratified by year, *i* (2006–2015); age group, *j* (50–54, 55–59, 60–64, 65–69 and 70–74 years); and sex, *k* (men, women). Let PFC*_i,j,k_*denote PFC in the whole of Sweden for year, *I*; age group, *j*; and sex, *k*. The expected number of colonoscopies in a county equals ∑(P*_i,j,k_*×PFC*_i,j,k_*) over all {*i, j, k*}.

## Results

In total, 305,378 colonoscopies were taken into account in this study. We excluded 1691 registered colonoscopies for Swedish-born patients with missing data for assessing educational level.

The PFC increased between 2006 and 2015 in all the regions (Table 1). In one of the regions, ROU, the estimated yearly PFC more than doubled, and in the other regions it almost doubled. Within the period 2006–2015, we estimated the largest PFC for the age group 65–69 years in the RSG. In contrast, in the other regions, the largest PFC was estimated for the age group 70–74 years. In all the regions, we estimated a higher PFC among the women compared to the men, but the 2006–2015 PFC difference between men and women was less pronounced in the RSG compared to the other regions. The PFC for the period 2006–2015 was highest in the group with a low educational level in all the regions – except in RN, where it was slightly lower compared to the intermediate and highly educated populations. There were no marked differences in the PFC for the period 2006–2015 between foreign-born and Swedish-born people – except in RN, where the highest PFC for 2006–2015 was among foreign-born people, and in RS, where the PFC for 2006–2015 was lowest among foreign-born people (see Table 1). There were differences in the observed-to-expected number of colonoscopies in the period 2006–2015 in each of the 21 counties in Sweden, where the expected number of colonoscopies was obtained from the year-, age- and sex-specific PFC for the total study population (Figure 1).

**Table 1.** Numbers of colonoscopies performed between 2006 and 2015 in people aged 50–74  from six Swedish regions and the corresponding population frequencies of colonoscopy.

	Region Stockholm-Gotland		Region West		Region South		Region South-East		Region Uppsala- Örebro		Region North	
	N	PFC	N	PFC	N	PFC	N	PFC	N	PFC	N	PFC
By year												
2006	4049	8.1	3500	8.3	3218	6.2	1454	5.1	4237	7.3	2288	8.7
2007	4468	8.7	4035	9.4	3674	7.0	1547	5.3	5634	9.6	2421	9.1
2008	5623	10.7	5105	11.7	4019	7.5	1540	5.2	6517	10.9	2719	10.1
2009	5801	10.7	5073	11.4	4296	7.9	1967	6.6	6713	11.1	2877	10.6
2010	6485	11.7	6081	13.4	5420	9.8	1932	6.4	7838	12.7	2969	10.8
2011	7114	12.6	6595	14.4	6714	11.9	2375	7.8	7721	12.4	3246	11.7
2012	7401	12.8	6573	14.0	6758	11.8	2336	7.6	7739	12.3	3334	12.0
2013	8136	13.8	7308	15.4	7007	12.1	2312	7.4	7720	12.1	3329	11.9
2014	8287	13.8	7820	16.2	8042	13.7	2610	8.3	7991	12.4	3992	14.1
2015	8032	13.2	7964	16.3	6484	10.9	2902	9.1	10,012	15.3	4063	14.3
By age (years)												
50–54	8772	6.6	8138	7.9	7567	6.2	2884	4.5	9366	7.3	4301	7.6
55–59	10,150	8.4	10,120	10.2	8979	7.5	3630	5.7	11,856	9.2	5375	9.2
60–64	16,297	13.7	13,532	13.6	12,300	10.0	4738	7.1	16,581	12.2	7186	11.9
65–69	18,219	16.9	14,724	16.1	14,272	12.2	5158	8.2	17,954	14.0	7564	13.6
70–74	11,958	15.3	13,506	19.2	12,514	13.8	4565	9.4	16,365	16.8	6798	15.5
By sex												
Men	31,432	11.2	27,581	11.8	25,682	8.9	9956	6.4	33,123	10.5	14,581	10.4
Women	33,964	11.6	32,439	13.8	29,950	10.2	11,109	7.1	38,999	12.4	16,652	12.0
By immigrant status/educational level												
Swedish-born/low	10,111	12.5	14,659	13.1	13,936	10.0	5944	7.0	18,195	11.8	6774	10.8
Swedish-born/intermediate	22,080	11.8	22,456	13.1	20,882	9.8	8336	6.7	29,379	11.4	14,540	10.9
Swedish-born/high	18,742	10.4	13,709	12.1	13,320	9.5	4572	6.8	15,706	10.8	7545	11.0
Foreign-born	14,463	11.1	9196	12.7	7494	8.9	2123	6.6	8842	11.7	2374	12.2

Note: PFC was estimated by marginal means from Poisson regression models with year, age, sex and immigrant status/education level as covariates (one model for each region).

N: no. of colonoscopies; PFC: population frequency of colonoscopy (no. of colonoscopies per 1000 per year).

The PFC in the RSG increased from 7.9 to 13.7 (men) and from 9.0 to 13.5 (women) between the pre- (2006–2007) and post- (2014–2015) implementation periods (Table 2). Furthermore, within the other five regions, the PFC estimates revealed marked increases over time for both men and women. Compared with the RSG, one region (RW) showed a systematically higher PFC, and two regions (RS and RSE) showed a systematically lower PFC (Table 2). The estimated pre–post increase in PFC (ΔPFC) within the RSG was (i) greater for men than for women (5.8 vs. 4.5) and (ii) smaller for people aged 70–74 than for those aged 60–69 (5.5 vs. 9.0), while the corresponding ΔPFCs within each of the other five regions were (i) not greater, or even smaller, for men and (ii) not smaller, or even larger, for elderly people aged between 70 and 74 (Table 2). We did not find systematically different patterns in ΔPFCs across immigrant status/education level in the RSG. The corresponding patterns varied across the other five regions (Table 2).

**Table 2.** Changes in population frequency of colonoscopy (PFC) in six Swedish regions between pre- (2006–2007) and post (2014–2015) implementation of an organised bowel cancer screening programme for people aged 60–69 living in one of the regions (Region Stockholm-Gotland).

	Region Stockholm-Gotland			Region West			Region South			Region South-East			Region Uppsala-Örebro			Region North		
	Pre	Post	Diff. (95% CI)	Pre	Post	Diff. (95% CI)	Pre	Post	Diff. (95% CI)	Pre	Post	Diff. (95% CI)	Pre	Post	Diff. (95% CI)	Pre	Post	Diff. (95% CI)
By age (years)																		
50–54	5.7	7.4	**1.6** **(1.2, 2.1)**	5.8	9.5	**3.7** **(3.1, 4.2)**	4.6	8.0	**3.4** **(2.9, 3.8)**	3.9	5.6	**1.6** **(1.1, 2.2)**	6.0	8.0	**2.1** **(1.6, 2.5)**	6.6	9.2	**2.6** **(1.9, 3.3)**
55–59	7.2	9.4	**2.2** **(1.7, 2.7)**	7.2	12.3	**5.0** **(4.4, 5.7)**	5.5	9.7	**4.2** **(3.7, 4.7)**	4.8	6.7	**1.9** **(1.3, 2.5)**	6.9	10.7	**3.7** **(3.2, 4.3)**	7.5	11.6	**4.1** **(3.3, 4.9)**
60–64	8.7	16.8	**8.1** **(7.5, 8.8)**	9.2	19.4	**10.2** **(9.5, 11.0)**	6.7	14.1	**7.5** **(6.9, 8.0)**	5.3	10.7	**5.4** **(4.7, 6.1)**	8.5	16.9	**8.5** **(7.9, 9.1)**	8.9	17.7	**8.8** **(7.8, 9.7)**
65–69	10.8	20.7	**9.9** **(9.2, 10.7)**	11.5	20.1	**8.6** **(7.7, 9.4)**	8.4	15.0	**6.7** **(6.0, 7.3)**	6.1	10.2	**4.1** **(3.3, 4.8)**	10.6	16.1	**5.4** **(4.8, 6.1)**	10.6	16.5	**5.9** **(4.9, 6.9)**
70–74	12.3	17.8	**5.5** **(4.6, 6.4)**	12.4	24.0	**11.6** **(10.6, 12.6)**	9.0	17.2	**8.3** **(7.5, 9.0)**	6.7	11.8	**5.1** **(4.3, 6.0)**	11.6	20.2	**8.5** **(7.8, 9.3)**	12.0	18.3	**6.2** **(5.1, 7.4)**
By sex																		
Men	7.9	13.7	**5.8** **(5.4, 6.2)**	8.0	15.1	**7.1** **(6.7, 7.6)**	6.1	11.5	**5.3** **(5.0, 5.7)**	5.0	8.5	**3.5** **(3.1, 4.0)**	7.8	12.8	**5.0** **(4.6, 5.4)**	8.2	13.4	**5.2** **(4.6, 5.7)**
Women	9.0	13.5	**4.5** **(4.1, 4.9)**	9.7	17.4	**7.7** **(7.3, 8.2)**	7.0	13.3	**6.3** **(5.9, 6.6)**	5.4	8.9	**3.4** **(3.0, 3.9)**	9.0	14.8	**5.8** **(5.5, 6.2)**	9.5	15.2	**5.7** **(5.1, 6.3)**
By immigrant status/education																		
Swedish-born/low	8.9	15.7	**6.9** **(6.1, 7.7)**	8.6	16.5	**7.8** **(7.2, 8.5)**	7.1	13.0	**6.0** **(5.4, 6.5)**	5.1	9.1	**3.9** **(3.3, 4.5)**	8.5	14.8	**6.3** **(5.7, 6.9)**	8.5	14.6	**6.1** **(5.2, 7.0)**
Swedish-born/intermediate	8.5	14.4	**5.9** **(5.4, 6.4)**	9.1	16.7	**7.6** **(7.1, 8.2)**	6.7	12.5	**5.9** **(5.5, 6.3)**	5.1	8.9	**3.8** **(3.3, 4.2)**	8.3	14.1	**5.8** **(5.4, 6.2)**	8.5	13.8	**5.3** **(4.8, 5.9)**
Swedish-born/high	7.3	12.3	**5.0** **(4.5, 5.5)**	8.7	15.7	**7.0** **(6.3, 7.6)**	6.4	12.6	**6.2** **(5.7, 6.8)**	5.6	8.5	**2.9** **(2.2, 3.5)**	8.4	12.6	**4.2** **(3.6, 4.7)**	8.6	14.3	**5.7** **(4.9, 6.5)**
Foreign-born	9.4	12.3	**2.9** **(2.3, 3.5)**	9.0	16.0	**7.0** **(6.2, 7.9)**	6.2	11.2	**5.0** **(4.3, 5.6)**	5.4	8.2	**2.7** **(1.8, 3.7)**	8.6	13.5	**4.9** **(4.2, 5.7)**	11.3	13.3	**2.0** **(0.4, 3.6)**

Note: The PFC was estimated by marginal means from Poisson regression models using period (pre-, post-implementation), age, sex and immigrant status/education level as covariates (one model for each region).

Differences in PFC (Diff.) were estimated via suitable parameterisations of Poisson regression models with period (pre, post-implementation), age, sex and immigrant status/education level as covariates (one model for each region).

Pre: pre-implementation period (no. per 1000/year); Post: post-implementation period (no. per 1000/year); Diff.: difference in PFC pre- and post-implementation; CI: confidence interval.

## Discussion

Using the available registry data on colonoscopy patients, we estimated that the PFC largely increased between 2006 and 2015 in all Swedish health-care regions. For a screening-relevant age group of 50–74 years, the estimated increase in the PFC after (2014–2015) vs. before (2006–2007) the implementation of the organised bowel cancer screening programme among inhabitants aged 60–69 in the RSG was (i) greater for men than for women and (ii) smaller for people aged 70–74 than for those aged 60–69. By contrast, within each of the other five health-care regions in Sweden, we estimated that the corresponding increases in the PFC were (i) not greater, or even smaller, for men and (ii) not smaller, or even larger, for elderly people aged between 70 and 74. Within the intervention region, the RSG, the pattern of estimated increases in the PFC across the population groups defined by immigrant status/educational level was not systematically different compared to the corresponding patterns in the other five regions.

The strength of this study is that it was nationwide, comparing an intervention region with five control regions. The large number of colonoscopies analysed and the fact that it appears unlikely that the ratio of registered/not registered colonoscopies *per region* differed substantially over the studied time period provided a robust base for conclusions. Swedish patient registers have high validity. For example, Ludvigsson et al.^21^ showed that the positive predictive value of the diagnoses in the Swedish National Inpatient Register has high validity, estimated at 85%–95%.

There were, however, limitations with this study. We estimated the proportion of screening colonoscopies in our dataset as 15% and hypothesised that this proportion would have an effect, explaining changes in sociodemographic disparities pre- vs. post implementation of the 2008 RSG CRC screening programme. We did not analyse changing patterns of colonoscopy indications overall during the period 2006–2015. This means that changes in sociodemographic disparities in colonoscopy frequency during the period may be associated with changes in indications other than CRC screening. Procedures performed by nurses were not registered, so data about nurse endoscopies are not available. This will be a considerable drawback in future studies comprising data after 2015; the number of nurse-performed endoscopies has increased markedly over the last few years. As at June 2020, there were, in total, 55 nurses performing endoscopies in Sweden. Until 2015, there were only a handful (personal communication with Ingrid Karström, Registered Nurse and Chairman of the Swedish Association of Swedish Endoscopy and Gastroenterology Personnel). In the county with the second-highest number of inhabitants, up to 50% of colonoscopies are not registered because organisations have large numbers of private health-care providers and local agreements. These major limitations could not be resolved in this retrospective study. Our sociodemographic variables were restricted to standard demographics (age, sex and residential region) and immigrant status/educational level. There are other variables of potential interest: employment status, marital status, and so forth. However, we highlight that educational level is a primary variable reflecting SES in relation to health outcomes. In Sweden, life expectancy at the age of 65 years is nearly three years longer in the population group with high education level than in the group with low educational level.^22^

Men have higher CRC incidence rates and mortality rates than women.^14^ Despite that, several studies have shown that uptake in FIT screening programmes is lower among men. A recent meta-analysis reported a significantly lower uptake of men vs. women (odds ratio: 0.84; 95% confidence interval: 0.75–0.95; *P* < 0.01).^15^ Our results indicate that the PFC was more pronounced for men after the implementation of the CRC screening programme in the RSG. In the first years of the RSG screening programme, guaiac-based FOBT was used, and FIT was introduced later. However, the latter had different cut-off levels for men (higher) and women (lower),^20^ and there is a possibility that our results reflect this difference. Another possibility is that men have more polyps^14^ and will therefore, to a larger extent, come back for a colonoscopy control within one to five years.

**Figure 2.**
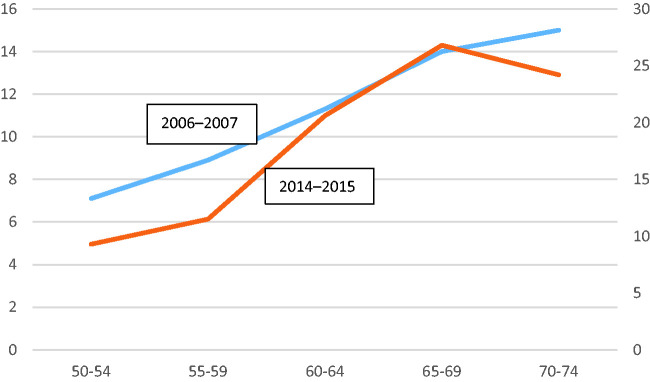
Population frequency of colonoscopy. X-axis, five-year age intervals; left-side Y-axis, 2006–2007; right-side Y-axis, 2014–2015.

The peak colonoscopy incidence was among people aged 65–69 post implementation, as opposed to 70–75 years pre-implementation (see Figure 2). The incidence of CRC is increasing in younger (<50 years) people.^23^,^24^ Notwithstanding, the majority of detected CRCs are among people in the age group 65–79 years.^23^,^25^ In the CRC pathway, there is a window of opportunity for secondary prevention, as a benign polyp takes approximately 10–20 years to develop into dysplasia and the initiation of a benign polyp occurs at about 30–60 years.^26^ From a CRC perspective, the peak colonoscopy incidence seen in the post-implementation RSG CRC screening period is, therefore, preferable. However, colonoscopy resources are limited, so the implementation of a general CRC screening programme may lead to crowding, where colonoscopies for people in other age groups and people with indications other than CRC are displaced in favour of colonoscopies for people invited for screening. We cannot disregard the possibility that such crowding may have been the case in the RSG and therefore suggest that the implementation of the RSG screening programme displaced colonoscopy resources from other age groups, as well as colonoscopies for indications other than CRC.

Only about half of the screening programmes worldwide collect data on participation by SES and ethnicity. Of these, 90% have reported lower participation among lower socioeconomic groups.^11^ On the contrary, our study provided no convincing data that the implementation of the CRC screening programme in the RSG altered PFC patterns in terms of patient educational status or whether a patient is Swedish-born or foreign-born. We believe this has to be further studied to support the hypothesis that the RSG CRC screening programme did not contribute to socioeconomic inequality in CRC treatment and mortality.

In summary, the implementation of a regional CRC screening programme lowered the peak PFC incidence from 70–74 years to 64–69 years, and we discussed this rationale from a CRC perspective. The expected post-implementation change^11^,^15^ in PFC socioeconomic patterns (sex, SES and ethnicity) was not evident in our data.
